# Clinical Features and Courses of Adenovirus Pneumonia in Healthy Young Adults during an Outbreak among Korean Military Personnel

**DOI:** 10.1371/journal.pone.0170592

**Published:** 2017-01-23

**Authors:** Ji Young Park, Bong-Joon Kim, Eun Jung Lee, Kwi Sung Park, Hee Sun Park, Sung Soo Jung, Ju Ock Kim

**Affiliations:** 1 Division of Pulmonary, Allergy and Critical Care Medicine, Department of Internal Medicine, Hallym University Sacred Heart Hospital, Anyang, Korea; 2 Division of Pulmonary and Critical Care Medicine, Department of Internal Medicine, The Armed Forces Daejeon Hospital, Daejeon, Korea; 3 Department of Internal Medicine, Kosin University College of Medicine, Busan, Korea; 4 Chungcheongnam-Do Institute of Health and Environment Research, Daejeon, Korea; 5 Division of Pulmonary and Critical Care Medicine, Department of Internal Medicine, Chungnam National University Hospital, Daejeon, Korea; Curtin University, AUSTRALIA

## Abstract

**Background:**

The number of pneumonia patients increased suddenly in Korean military hospitals in late December 2014, indicating the urgent need for an epidemic outbreak investigation.

**Methods:**

We conducted a prospective study of pneumonia etiology among immunocompetent young adults admitted to Daejeon Armed Forces hospital. Patient blood and sputum samples were subjected to conventional culture, serology, and polymerase chain reaction tests for respiratory viruses and atypical pathogens.

**Results:**

From January to May 2015, we enrolled 191 (189 male) adults with pneumonia; the mean age was 20.1 ± 1.3 years. Five patients had severe pneumonia, and one died. Pathogenic human adenoviruses were most common (HAdV, 153/191 [80.1%]), indicating a HAdV pneumonia outbreak. Genotyping of 35 isolates indicated that 34 matched HAdV-55 and one matched HAdV-2. HAdV pneumonia infected recruit trainees most frequently. High and prolonged fever, nasal congestion, sore throat, and pharyngeal inflammation were significantly more common in the HAdV pneumonia group, compared to patients with other or unknown causes of pneumonia. Only 12% of HAdV pneumonia patients displayed leukocytosis, whereas febrile leukopenia (62.7%) and thrombocytopenia (41%) were commonly observed. HAdV pneumonia patient chest CT scans displayed ground glass opacity (with or without septal thickness) with consolidation in 50.0% of patients.

**Conclusions:**

An outbreak of HAdV respiratory infection occurred at the Korean military training center. HAdV pneumonia exhibited specific laboratory and clinical features, and although most patients were cured without complication, some progressed to respiratory failure and fatality. Therefore, HAdV vaccine should be provided to military trainees in Korea.

## Introduction

Viral respiratory infection is particularly important in military populations who experience overexertion, psychological stress, and crowding within confined spaces [1]. There are many reports of respiratory virus outbreaks in the military [1–3]. In many settings, human adenovirus (HAdV) was the main causative pathogen and occasionally led to death [3, 4]. HAdVs are also important in the pathogenesis of community acquired pneumonia (CAP) among both immunocompetent and immunocompromised individuals [5, 6]. Although despite reports of a low prevalence in previous studies (1.4–4%), adenovirus-related CAP was recently ranked in the top 10 etiologies of CAP by larger studies [7, 8]. Moreover, HAdV is easily transmittable and can be highly contagious [9]. The clinical features of respiratory adenoviral infection among military personnel were described previously; however, HAdV pneumonia in immunocompetent individuals and risk factors of disease progression to severe pneumonia or acute respiratory failure have not been well studied.

In December 2014, a sudden increase in patients with febrile respiratory illness and pneumonia occurred among military hospitals of the South Korean Army, including our institution. Medical staff noted that the rates of HAdV-positive respiratory specimens had also increased. Therefore, we deduced an emergent HAdV outbreak and aimed primarily to identify the pathogenic agent(s) causing the sudden increase in pneumonia cases. We also aimed to describe the clinical features and radiological findings of HAdV pneumonia in immunocompetent individuals.

## Methods

### Study population and design

We conducted a prospective study of CAP in immunocompetent military trainees or active duty soldiers admitted for pneumonia to Daejeon Armed Forces Hospital, South Korea from January to May 2015. Patients are referred to this 500-bed hospital by basic and advanced military training centers and other military hospitals. In South Korea, military service is mandatory for all healthy men ≥18 years old. Trainees spend 6 weeks on basic military training, and then proceed to advanced training centers or active duty [10]. All military trainees or active duty members, but not officers, were eligible for enrollment if they were ≥18 years old and had been admitted to the study hospital for pneumonia, defined by acute respiratory symptoms (fever, cough, sputum, dyspnea, and pleuritic chest pain) and pulmonary infiltrates on chest X-rays or computed tomography (CT) scans. Patients diagnosed with other pulmonary diseases were excluded.

### Ethical considerations

This study was conducted in accordance with the amended Declaration of Helsinki. The study protocol was approved by the Institutional Review Board of the Armed Forces Medical Command (AFMC 15016-IRB-15-011), and informed consent was obtained from all patients. Consent was verbal in nature because an etiological evaluation of pneumonia is considered routine care. The verbal consent procedure was approved by the Ethics Committee of the Armed Forces Medical Command. The study medical officer maintained a register of patients who consented verbally to participate in the cohort.

### Data collection

Patient sputum samples for conventional culture and polymerase chain reaction (PCR) tests and blood samples for culture and serologic tests were collected before prescribing medications, which were chosen at the physicians’ discretion. Outpatient clinic or emergency room investigating physicians collected clinical information. Sputum specimens were collected from all patients at enrollment. These were acceptable for culture if they satisfied Murray–Washington classification degrees IV or V [11]. Sputum specimens were cultured routinely and tested by PCR. Severe pneumonia was defined by one or more of the following criteria: 1) invasive mechanical ventilation, 2) use of vasopressors, 3) >50% of lung parenchymal involvement. All cases were scored according to the pneumonia severity index (PSI) and CURB-65 [12, 13].

### Multiplex real-time PCR

We performed multiplex PCR for human respiratory viruses using the AdvanSure^™^ RV real-time PCR Kit (LG Life Sciences, Korea; Supplementary Methods). This assay targets 12 types of pathogenic RNA viruses: rhinoviruses A/B/C, influenza viruses A/B, coronaviruses 229E/NL63/OC43, respiratory syncytial viruses A/B, parainfluenza viruses 1/2/3, and metapneumovirus; and two types of DNA viruses: adenovirus and bocavirus [14].

We performed multiplex PCR for the respiratory bacterial pathogens *Mycoplasma pneumoniae*, *Chlamydophila pneumoniae*, *Legionella pneumophila*, and *Bordetella pertussis*, using the Seeplex^®^ PneumoBacter ACE Detection assay (Seegene, Seoul, Korea; Supplementary Methods). *Streptococcus pneumoniae* and *Haemophilus influenzae* were not analyzed because we could not differentiate true infection from colonization of these pathogens [15]. We performed nested PCR in the hypervariable region of the hexon gene for genotyping using previously described nested PCR conditions and primer sequences for hexon gene amplification [16]. PCR products were purified with the QIA quick PCR purification kit (Qiagen, Valencia, CA, USA) prior to their use as nucleotide sequencing templates. The genotype of each isolate was determined according to the serotype of the highest scoring strain in Genbank, using the Basic Local Alignment Search Tool (BLAST).

### Data analysis

Categorical variables were analyzed by the chi-squared or Fisher’s exact test. Continuous variables were compared by the Student’s *t*-test or Mann–Whitney U-test. A *p*-value <0.05 was considered statistically significant. All analyses were performed using SPSS for Windows ver. 18.0 (SPSS, Chicago, IL, USA).

## Results

### Baseline characteristics

**Fig 1:**
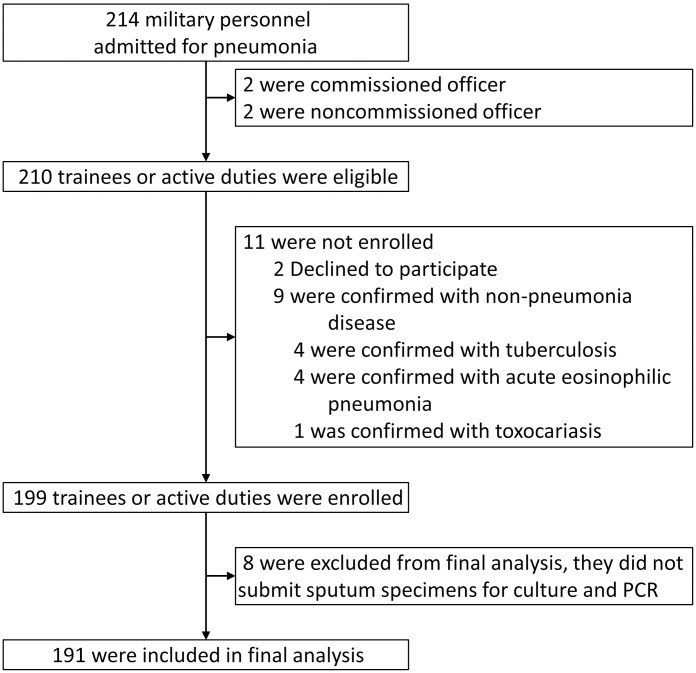
Enrollment of patients with community acquired pneumonia in the Korean military.

Of 210 eligible patients, 199 were enrolled (Fig 1): two patients refused enrollment, and nine others had tuberculosis (n = 4), acute eosinophilic pneumonia (n = 4), or toxocariasis (n = 1). Eight cases were excluded because of missing sputum samples. Therefore, 191 patients were included in the final analysis. The mean age was 20.1 ± 1.3 years; most patients were men (99.0%), the current smoking rate was 32.3%, and 94.8% of patients had received the seasonal (2014–15) influenza vaccine. Excepting one patient who was incidentally diagnosed with Gitelman’s syndrome, none had systemic disease, in accordance with the requirement for all military trainee candidates to pass the medical conscription examination (Table 1).

**Table 1:** Clinical characteristics of 191 patients with community-acquired pneumonia in the military.

Variables	Adenovirus positive (n = 153)	Adenovirus negative (n = 38)	Total (n = 191)	*P* value
Mean age, years	20.1 ± 1.3	20.2 ± 1.0	20.1 ± 1.3	0.930
Sex, male	151 (98.7)	38 (100)	189 (99.0)	1.000
Median service period, weeks, (IQR)	7.0 (5.9–8.7)	8.6 (6.4–30.9)	7.1 (5.9–9.0)	0.032
Current smoker	48 (31.2)	14 (36.8)	62 (32.3)	0.503
Influenza vaccine (<1 year)	146 (94.8)	36 (94.7)	182 (94.8)	0.986
Asthma	10 (6.5)	3 (7.9)	13 (6.8)	0.724
Symptoms and signs				
Cough	150 (98.0)	38 (100)	188 (98.4)	1.000
Fever	151 (98.7)	33 (86.8)	185 (96.4)	0.004
Maximal temperature	39.3 ± 0.9	38.0 ± 1.2	39.1 ± 1.1	<0.001
High fever (≥40.0°C)	46 (30.1)	3 (7.9)	49 (25.7)	0.004
High fever (≥39.0°C)	116 (75.8)	9 (23.7)	125 (65.4)	<0.001
Duration of fever, days	6.3 ± 1.7	5.3 ± 1.9	6.1 ± 1.8	0.002
Sputum production	141 (92.2)	33 (86.8)	174 (91.1)	0.297
Purulent sputum	133/141 (94.3)	30/33 (90.9)	163/174 (93.7)	0.439
Rhinorrhea	107 (69.9)	24 (63.2)	131 (68.6)	0.421
Nasal congestion	96 (62.7)	16 (42.1)	112 (58.6)	0.021
Throat clearing	96 (62.7)	15 (39.5)	111 (58.1)	0.009
Sore throat	115 (75.2)	11 (28.9)	126 (66.0)	<0.001
Pharyngeal inflammation	133 (86.9)	18 (47.4)	151 (79.1)	<0.001
Blood-tinged sputum	47 (30.7)	6 (15.8)	53 (27.7)	0.066
Dyspnea or chest discomfort	32 (20.9)	12 (31.6)	44 (23.0)	0.162
Chest pain	27 (17.6)	10 (26.3)	37 (19.4)	0.226
Headache	109 (71.2)	19 (50.0)	128 (67.0)	0.013
Diarrhea	31 (20.3)	3 (7.9)	34 (17.8)	0.074
Myalgia	90 (58.8)	19 (50.0)	109 (57.1)	0.325
Wheezing	2 (1.3)	2 (5.3)	4 (2.1)	0.176
Crackle	81 (52.6)	19 (50.0)	100 (52.1)	0.774
Systemic blood pressure, mm Hg	124.7 ± 13.7	125.8 ± 14.1	124 ± 13.8	0.660
Heart rate, beats/min	93.9 ± 15.6	90.3 ± 16.7	93.2 ± 15.9	0.221
Respiratory rate, breaths/min	18.6 ± 4.1	18.4 ± 4.0	18.5 ± 4.1	0.839
Oxygen saturation in room air, %	97.8 ± 2.2	97.9 ± 1.3	97.8 ± 2.0	0.751

IQR: interquartile range

### Pathogen detection

Respiratory pathogens were detected in 183/191 (95.8%) patients. Using the multiplex PCR test, viruses were identified in 167/191 (87.4%) patients (Table 2). The most common pathogen was adenovirus [HAdV, 153/191 (80.1%)], suggesting a HAdV pneumonia outbreak. HAdV genotyping determined that 34/35 samples (97.1%) were HAdV-55 infected, whereas 1/35 (2.9%) was HAdV-2 infected. PCR tests of bronchial washing or bronchoalveolar lavage samples detected HAdV in 5/18 (27.8%) patients and *M*. *pneumoniae* in 3/18 (16.7%) patients (Table 2). Typical bacteria (*Klebsiella pneumoniae*) were cultured by conventional culture in only one patient. Cases involving co-infection with multiple pathogens are listed in S1 Table.

**Table 2:** Distribution of viral and bacterial pathogens in cases of community-acquired pneumonia in the military.

Etiologic agents (isolated patients)	No. (%) of positive findings	PCR from sputum or bronchial washing	PCR from bronchial washing	Serology	Culture	Urine antigen assay
Viral pathogens (n = 191)	167 (87.4)	167/191	7/18	-	-	-
Adenovirus	153 (80.1)	153/191	5/18	-	-	-
Rhinovirus	29 (15.2)	29/191	2/18	-	-	-
Coronavirus	13 (6.8)	13/191	0/18	-	-	-
Human metapneumovirus	11 (5.8)	11/191	0/18	-	-	-
Influenza A or B	7 (3.7)	7/191	1/18	-	-	-
Parainfluenza virus	5 (2.6)	5/191	1/18	-	-	-
Respiratory syncytial virus	4 (2.1)	4/191	0/18	-	-	-
Bocavirus	1 (0.5)	1/191	0/18	-	-	-
Atypical bacterial pathogens						
*Mycoplasma pneumoniae*	17 (8.9)	11/170	3/18	7/170	-	-
*Chlamydophila pneumoniae*	6 (3.1)	0/170	0/18	6/170	-	-
*Legionella pneumophila*	0	0/170	0/18	-	-	0/182
*Bordetella pertussis*	0	0/170	0/18	-	-	-
Typical bacterial pathogens*						
*Streptococcus pneumoniae*	0	-	-	-	0/139	0/182
*Klebsiella pneumoniae*	1 (0.5)	-	-	-	1/139	-

*In patients with bronchial washing fluid or acceptable sputum, defined as satisfying Murray–Washington classification degree IV or V (n = 139)

### HAdV pneumonia outbreak epidemiology

**Fig 2:**
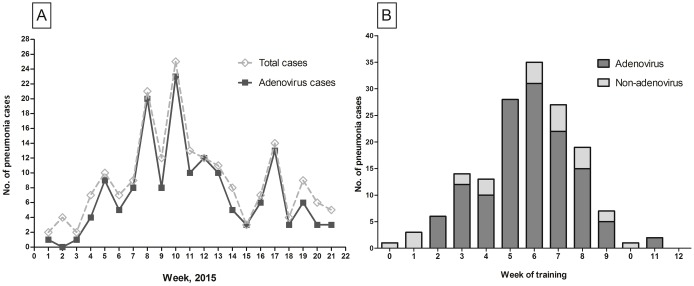
(A) Numbers of patients with community acquired pneumonia and adenovirus pneumonia by week during January–May 2015. (B) Number of patients with adenovirus pneumonia (HAdV) and non-adenovirus pneumonia by military training week among newly recruited trainees (≤12 weeks of training or service period).

The number of weekly HAdV-positive cases was plotted (Fig 2A). HAdV cases peaked at week 10 (early March 2015). A secondary peak occurred at week 17. HAdV pneumonia cases occurred throughout the training period but were most common around the end of basic training (week 6, Fig 2B). Most HAdV pneumonia patients were basic military trainees or personnel who had recently completed training; active duty service personnel were not usually affected, even during outbreak peaks. There were no significant differences in the mean age, smoking status, and influenza vaccination rate (Table 1). The median military service period (including training period) was shorter in the HAdV-positive pneumonia group than in the negative group (7.0 vs. 8.6, *p* = 0.032).

### HAdV pneumonia clinical characteristics

We compared clinical characteristics according to the HAdV PCR test results (Tables 1 and 3). Fever, high fever (≥39.0°C), nasal congestion, sore throat, throat clearing, headache, and pharyngeal inflammation were more common among HAdV pneumonia patients than in others. Many hematologic findings of the HAdV pneumonia group differed significantly from those of the negative group (Table 3), including leukocytosis, febrile leukopenia, thrombocytopenia, and hematocrit. The mean serum levels of C-reactive protein (CRP) and procalcitonin were not significantly different between the two groups. Pleural fluid analysis was available for three HAdV pneumonia patients, and their samples were found to be lymphocyte dominant (mean, 76.1%) with a mean adenosine deaminase level was 61.9 IU/L. The mean PSI score was higher among the HAdV-positive group than the negative group (29.8 vs. 24.8 *p* = 0.005) and PSI class rates tended to be higher among the HAdV-positive group (*p* = 0.046, Table 4).

**Table 3:** Laboratory findings of 191 patients with community-acquired pneumonia in the military.

Variables	Adenovirus positive (n = 153)	Adenovirus negative (n = 38)	Total (n = 191)	P value
White blood cell (WBC)				
WBC, 10^9^/L, admission day	6.48 ± 3.31	10.46 ± 4.74	7.27 ± 3.96	<0.001
Leukopenia (<4×10^9^/L)	35 (22.9)	1 (2.6)	36 (18.8)	0.002
Leukocytosis (>10×10^9^/L)	19 (12.4)	16 (42.1)	35 (18.3)	<0.001
Neutrophil, %	67.6 ± 12.5	74.8 ± 9.4	69.0 ± 12.3	0.001
Lymphocyte, %	21.5 ± 10.7	16.8 ± 7.2	20.6 ± 10.3	0.002
Leukopenia during febrile period	96 (62.7)	7 (18.4)	103 (53.9)	<0.001
WBC, 10^9^/L, nadir (n = 103)	3.15 ± 0.53	3.18 ± 0.54	3.15 ± 0.53	0.884
Days from fever onset to WBC nadir (n = 103)	6.6 ± 1.6	6.7 ± 0.5	6.6 ± 1.5	0.817
Days from fever onset to the most radiologic aggravation	6.4 ± 1.7	5.8 ± 1.9	6.3 ± 1.8	0.072
Platelet				
Platelet, 10^9^/L, admission day	179 ± 65	242 ± 73	192 ± 71	<0.001
Thrombocytopenia (<150×10^9^/L)	55 (35.9)	2 (5.3)	57 (29.8)	<0.001
Thrombocytopenia during febrile period	64 (41.8)	3 (7.9)	67 (35.1)	<0.001
Hematocrit, %	40.8 ± 2.9	42.1 ± 3.1	41.1 ± 2.9	0.020
CRP, mg/dL	6.95 ± 4.85	7.25 ± 6.40	7.01 ± 5.18	0.747
Procalcitonin, ng/mL (n = 149)	0.35 ± 0.90	0.31 ± 1.02	0.34 ± 0.92	0.820
pH	7.39 ± 0.04	7.38 ± 0.04	7.39 ± 0.04	0.591
BUN, mg/dL	11.1 ± 3.4	12.7 ± 3.7	11.4 ± 3.5	0.015
Sodium, mmol/L	135 ± 11	137 ± 2	136 ± 3	0.225
Glucose, mg/dL	106 ± 19	101 ± 13	104 ± 17	0.218
Creatine phosphokinase, IU/L (n = 158)	778 ± 977	462 ± 637	716 ± 927	0.090
AST, IU/L	49 ± 47	38 ± 34	47 ± 45	0.186
ALT, IU/L	33 ± 37	30 ± 29	33 ± 35	0.725

CRP: C-reactive protein, BUN: blood urea nitrogen, AST: aspartate aminotransferase, ALT: alanine aminotransferase

**Table 4:** Pneumonia severities and outcomes of 191 patients with community-acquired pneumonia in the military.

Variables	Adenovirus positive (n = 153)	Adenovirus negative (n = 38)	Total (n = 191)	P value
Pneumonia severity on admission				
PSI score	29.8 ± 13.2	24.8 ± 8.3	28.7 ± 12.7	0.005
PSI class				0.046
Class I	102 (66.7)	33 (86.8)	135 (70.7)	
Class II	48 (31.4)	5 (13.2)	53 (27.7)	
Class III	3 (2.0)	0 (0)	3 (1.6)	
Class IV–V	0 (0)	0 (0)	0 (0)	
CURB-65 score				0.420
0 point	146 (95.4)	35 (92.1)	181 (94.8)	
1 point	7 (4.6)	3 (7.9)	10 (5.2)	
2–5 points	0 (0)	0 (0)	0 (0)	
Outcomes				
Hospital days	9.2 ± 4.5	9.3 ± 4.4	9.2 ± 4.5	0.947
Complications				
Acute heart failure	2 (1.3)	0	2 (1.0)	1.000
Central vein thrombosis	1 (0.7)	0	1 (0.5)	1.000
Delirium	1 (0.7)	0	1 (0.5)	1.000
ICU admission	5 (3.3)	1 (2.6)	6 (3.1)	1.000
Mechanical ventilation	2 (1.3)	0	2 (1.0)	1.000
Extra corporeal membrane oxygenation	1 (0.7)	0	1 (0.5)	1.000
Mortality	1 (0.7)	0	1 (0.5)	1.000

PSI: pneumonia severity index, ICU: intensive care unit. All cases were scored according to the pneumonia severity index (PSI) and CURB-65 [12, 13].

### Radiological comparisons

**Fig 3:**
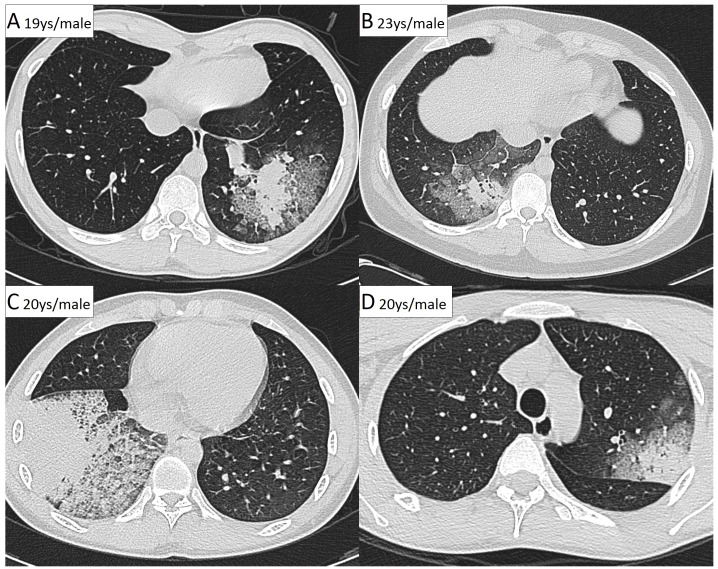
(A–D) Computed tomography scans of four patients with adenoviral pneumonia. Parenchymal opacities (consolidation and ground-grass opacity) with septal thickness were distributed along bronchovascular bundles or in the subpleural lung.

Chest CT images were obtained from all patients except one. Ground-glass opacity (GGO; an area of increased opacity without obscuration of the underlying vessels) and septal thickening were more common in the HAdV-positive group than in the negative group (*p* = 0.014 and *p* = 0.001, respectively). Nodules and bronchial wall thickening were less common in the HAdV pneumonia group. The parenchymal opacities in HAdV-positive pneumonia patients more commonly involved the lower lobes. The mean number of involved lung lobes was significantly different between the HAdV-positive and negative groups (1.4 lobes vs. 2.0 lobes, *p* = 0.032). Among HAdV pneumonia patients, the most common CT pattern was segmental GGO with central consolidation (50%, S2 Table and Fig 3).

### Severe HAdV pneumonia

Among HAdV pneumonias, severe pneumonia was observed in five patients. Severe pneumonia patients and others did not differ significantly with respect to demographic characteristics and most symptoms (S3 Table). However, high fever, dyspnea, and chest discomfort were more frequent and febrile periods were significantly longer among patients with severe HAdV pneumonia, compared to others (8.6 ± 1.9 vs. 6.3 ± 1.6 days; *p* = 0.002). The time from fever onset to the greatest radiologic aggravation (increased opacity on follow-up chest X-ray) was also longer in the severe group (9.0 ± 2.7 vs. 6.3 ± 1.6, *p* = 0.001). Severe HAdV pneumonia was associated with a lower white blood cell count (WBC) and platelet count on admission day (*p* <0.001 and *p* = 0.042, respectively). Although the mean CRP value was higher in the severe group (*p* = 0.002), the mean serum procalcitonin concentration did not differ significantly between patients with severe pneumonia and others (*p* = 0.102; S4 Table).

### Clinical outcomes

There were some complications among HAdV pneumonia patients. Acute heart failure occurred in two patients. Delirium occurred in one patient. One patient developed upper-extremity deep vein thrombosis, possibly resulting from a central line catheter. Two patients required mechanical ventilation. Of these latter patients, one expired from respiratory and heart failure (Table 4).

## Discussion

Our results show that an outbreak of HAdV pneumonia occurred in Korean military training centers and indicate that emergent-type HAdV-55 infections might have caused the outbreak. In this outbreak, HAdV pneumonia was associated with specific clinical symptoms, laboratory results, and chest CT scan findings.

Recently, multiple outbreaks of acute respiratory disease have been associated with an emergent variant, HAdV-55 (formerly named HAdV-11a). Whole-genome sequencing of this variant indicates potential hexon gene recombination between the HAdV-11 and HAdV-14 strains [17]. Such outbreaks occurred in the military forces of Turkey in 2004 [18] and Singapore in 2005 [19]. In China, several outbreaks of HAdV-55 respiratory disease have been reported not only in the military, but also in the community (e.g., a senior high school in 2006 [20], Beijing in 2011 [21], military in 2012 [1], and a physical training facility in 2013 [22]). Since 2012, HAdV-55 has also been identified among severe pneumonia patients at a Korean military hospital [23].

Most HAdV pneumonia patients were basic trainees or personnel who finished their training recently. These results were similar to those of previous studies [24]. It is well documented that the most significant factor leading to HAdV infection and disease is a lack of preexisting, type-specific immunity against HAdV-4 and HAdV-7. Moreover, anti-HAdV4 immunity provided 60% protection from adenovirus-related hospitalization and 98% protection from infection [1, 25]. In the Korean military, it is not certain why active duty service personnel were infected less frequently than trainees. One possibility is that the former group may have had preexisting HAdV-55 antibodies from previous exposure. Another possibility is that the former group had other cross-protective HAdV antibodies. In 2006, HAdV-7 was the dominant type of HAdV in the Korea military [26]. HAdV-7 is in the same species group as HAdV-55 (HAdV-7/11/14/55 are in species group B) [27]. When a HAdV-14 outbreak occurred at U.S. military training facilities, preexisting HAdV-7 neutralizing antibodies provided cross-protection against HAdV-14 in recruits [28]. Other predisposing factors that might be associated with selective infection of newly recruited trainees include overcrowding and physical or emotional stress [29, 30].

HAdV pneumonia diagnoses began in the 2nd week of training and peaked at week 6, in close accordance with previous U.S. army studies. Kolavic-Gray et al. [25] found that HAdV-4-related acute respiratory diseases among military trainees peaked during week 5 of training, and Tate et al. [28] reported that HAdV-14-associated febrile respiratory illness peaked during weeks 4–6. One possible reason for this timing pattern is that sufficient numbers of infected or colonized trainees may be required to cause an outbreak [28]. Moreover, the 6-week training period is epidemiologically important because trainees usually move to advanced training centers or into active duty after completing basic training, possibly creating a secondary outbreak.

Our prospective analysis demonstrated that this HAdV pneumonia was associated with specific clinical features that differed from other pneumonias. Although the mean fever duration in HAdV pneumonia patients was similar to those in previous studies (6–9 days) [21, 31, 32], patients with HAdV pneumonia had higher and longer fevers than the HAdV-negative group. Moreover, the fever duration associated with severe HAdV pneumonia was prolonged relative to that of mild-moderate pneumonia, again corroborating previous reports [32]. Patients with HAdV pneumonia also had more upper respiratory tract symptoms than those in the negative group, suggesting that HAdV infected or colonized the upper respiratory tract first, then progressed to a lower respiratory tract infection. The most typical laboratory findings of HAdV pneumonia were febrile leukopenia and thrombocytopenia. In agreement with our results, Vento et al. [33] demonstrated lower WBC and platelet counts in a HAdV-14 pneumonia among U.S. military trainees. Patients with severe pneumonia had fewer WBC and lower platelet counts than those with mild to moderate pneumonia. Moreover, the mean times from fever onset to the nadir of WBC and to the greatest radiologic aggravation were similar and correlated (r = 0.513, *p* <0.001). These findings suggest that hematologic parameters might be used to monitor HAdV pneumonia and to index risk factors for disease progression. Finally, although the CRP levels were moderately elevated in our study, the mean procalcitonin level was only 0.35 μg/L, confirming that a bacterial infection was unlikely [34]. In fact, bacterial co-infection was rare. Among the 153/191 adenovirus-positive patients, only 3.9% (6/153) exhibited evidence of bacterial infection (typical: 1; atypical: 5). In contrast, among the 38/191 adenovirus-negative patients, 47.4% (18/38) had bacterial infections (p <0.001). This further confirms the HAdV infection findings in our cohort.

Very few case studies of the CT findings of HAdV pneumonia exist in the literature [35]. The most common pattern in our study was GGO with or without septal thickness and central consolidation, a finding specific to HAdV pneumonia that was not observed with HAdV-negative pneumonia. Our CT scans also demonstrated that single lobe involvement was more common than multi-lobe involvement, in contrast to a previous case report that suggested such cases usually manifested as bilateral involvement [35]. These observations might be useful during the differential diagnosis of young adults presenting with pneumonia symptoms during an outbreak; however, further radiological studies are needed.

No antiviral agents have been approved to treat HAdV pneumonia, and only limited data on the clinical responses of immunocompromised patients to cidofovir are available. However, Kim et al. [23], in another study of severe HAdV infection in the Korean military, suggested that early treatment with cidofovir should be considered for respiratory failure due to HAdV pneumonia. Most patients in our study were treated empirically with antibiotics, and most HAdV pneumonia cases were self-limiting without an antiviral agent. However, cidofovir was administered at a 5-mg/kg weekly dose to two severe HAdV pneumonia patients. One patient recovered without sequelae, whereas the other patient died. It would be unsafe to offer cidofovir to all HAdV pneumonia patients because of the risk of renal toxicity. Moreover, this drug is expensive and is not stocked regularly in hospitals. In this respect, the early identification of patients who might develop severe pneumonia is most important when selecting cases that will receive antiviral treatment [23]. Interestingly, the PSI scores and CURB-65 values were higher in the severe pneumonia group, whereas the absolute scores in this group did not indicate high risk. Notably, age is an important factor in these pneumonia severity score systems; accordingly, the lower scores obtained in our study might be partly attributable to the young ages of our adult patients. In addition, that these scoring systems were validated in communities where the main pathogens were bacterial, rather than viral [36]. Similar results were observed in pandemic H1N1 influenza pneumonia studies in which PSI and CURB-65 failed to predict admissions to intensive care or the need for mechanical ventilation [37, 38].

Our study has one limitation. Because pneumonia increased abruptly in our patients, we were uncertain of which pathogen(s) caused the outbreak and did not plan to conduct HAdV genotyping at the study outset. We only sent partially banked sputum specimens for HAdV genotyping confirmation after realizing that there a HAdV outbreak was occurring in the Korean military. It is uncertain whether vaccines against types 4 and 7, which are currently only available in the U.S. Army, will effectively protect against HAdV type 55. Therefore, new, more suitable vaccines should be developed for subtypes responsible for current outbreaks.

In conclusion, this HAdV pneumonia exhibited specific clinical and laboratory features and chest CT findings. Although the pneumonia severity varied, this infection can induce morbidity and fatality. A respiratory virus surveillance system and epidemiological expertise are urgently needed and HAdV vaccination should be considered in Korean military training centers.

## Supporting Information

S1 File Supplementary Methods. (DOCX)

S1 Table The distribution of co-infection with multiple pathogens. (DOCX)

S2 Table Chest computed tomography (CT) scan patterns of adenoviral pneumonia and number of days from fever onset to CT scan (n = 152). (DOCX)

S3 Table Comparison of clinical features associated with severe adenovirus pneumonia and mild to moderate adenovirus pneumonia among military personnel. (DOCX)

S4 Table Comparison of laboratory findings and outcomes associated with severe adenovirus pneumonia and mild to moderate adenovirus pneumonia among military personnel. (DOCX)

## Abbreviations

CAP: community acquired pneumonia
CRP: C-reactive protein
HAdV: Human adenovirus
PCR: polymerase chain reaction
PSI: pneumonia severity index
