# Supplementary material for: Clinical Features and Courses of Adenovirus Pneumonia in Healthy Young Adults during an Outbreak among Korean Military Personnel
Source: PLoS One. 2017 Jan 23;12(1):e0170592. doi: 10.1371/journal.pone.0170592 (PMC5256920; doi:10.1371/journal.pone.0170592)
Supplement: S1 File — (DOCX) [file pone.0170592.s001.docx]

**SUPPLEMENTARY INFORMATION**

**S1 File; Supplementary Methods**

**Viral Nucleic Acid Purification**

Nucleic acids (RNAs and DNAs) were extracted from 200 μL of sputum or bronchial washing samples using the TANBead® Viral Auto Plate system (Taiwan Advanced Nanotech Inc., Taoyuan City, Taiwan), according to the manufacturer’s protocol [1].

**Viral Multiplex Real-time PCR**

Five μL of primer probe mix and 10 μL of one-step RT-PCR premix were combined in a tube, followed by 5 μL of extracted nucleic acid. For the reverse transcription step, this mixture was incubated at 50°C for 10 min. Denaturation followed at 95°C for 30 sec, and then amplification for 10 cycles of PCR (15 sec at 95°C, 30 sec at 53°C, and 30 sec at 60°C), and 30 additional cycles of PCR for the detection of fluorescence signals (15 sec at 95°C, 30 sec at 53°C, 30 sec at 60°C). As an internal control, the human RNase P gene was quantified in all assayed samples [2].

**Bacterial DNA Purification**

Samples were extracted by an automated extraction procedure with the MagNA Pure LC DNA Isolation Kit (Roche, Indianapolis, IN, USA), according to the manufacturer’s instructions.

**Bacterial Multiplex PCR**

PCR amplification was performed in a total volume of 20 μL containing 3 μL of a nucleic acid sample and 17 μL PCR Mastermix (4 μL 5X PneumoBacter primers, 3 μL 8-methoxypsoralen, and 10 μL 2X Multiplex Master Mix) as per the manufacturer’s protocol. The PCR reaction underwent 40 cycles of amplification consisting of denaturation at 94°C for 0.5 min, annealing at 60°C for 1.5 min, and elongation at 72°C for 1.5 min [3]. The PCR target genes were, *M. pneumoniae*, *ITS1*; *C. pneumoniae*, *ompA*; *L. pneumophila*, *mip*; and *B. pertussis*, *prn* [4].

**Serological tests for atypical pathogens**

*M. pneumoniae* and *C. pneumoniae* infection were diagnosed if the presence of the microbe had been documented by either a positive serological test or a positive PCR assay. Enzyme immunoassay (Bio-Rad, Hercules, CA, USA) was used to identify *M. pneumoniae* and a microimmunofluorescence test (Vircell, Granada, Spain) to identify *C. pneumoniae*, according to the manufacturer's instructions. Serological diagnosis was based on a demonstration of seroconversion, defined as a change from a negative acute sample to a positive convalescent sample or a 4-fold rise in antibody titers between the paired samples. In patients for whom only an acute phase sample was available, infections were defined to be above a specific titer of IgM. Urine antigen tests for *Pneumococcus* and *Legionella* were performed with an immunochromatographic BinaxNOW® assay (Binax Inc., Scarborough, ME, USA), following the manufacturer’s protocol.

**Reference**

[1] C.H. Cho, C.K. Lee, M.H. Nam, S.Y. Yoon, C.S. Lim, Y. Cho, Y.K. Kim, Evaluation of the AdvanSure real-time RT-PCR compared with culture and Seeplex RV15 for simultaneous detection of respiratory viruses, Diagn Microbiol Infect Dis 79 (2014) 14-8.

[2] I. Rheem, J. Park, T.H. Kim, J.W. Kim, Evaluation of a multiplex real-time PCR assay for the detection of respiratory viruses in clinical specimens, Ann Lab Med 32 (2012) 399-406.

[3] M.C. Cho, H. Kim, D. An, M. Lee, S.A. Noh, M.N. Kim, Y.P. Chong, J.H. Woo, Comparison of sputum and nasopharyngeal swab specimens for molecular diagnosis of Mycoplasma pneumoniae, Chlamydophila pneumoniae, and Legionella pneumophila, Ann Lab Med 32 (2012) 133-8.

[4] C.L. Jung, M.A. Lee, W.S. Chung, Clinical Evaluation of the Multiplex PCR Assay for the Detection of Bacterial Pathogens in Respiratory Specimens from Patients with Pneumonia, Korean J Clin Microbiol 13 (2010) 40-46.
