# Supplementary material for: Clinical Features and Courses of Adenovirus Pneumonia in Healthy Young Adults during an Outbreak among Korean Military Personnel
Source: PLoS One. 2017 Jan 23;12(1):e0170592. doi: 10.1371/journal.pone.0170592 (PMC5256920; doi:10.1371/journal.pone.0170592)
Supplement: S1 Table — (DOCX) [file pone.0170592.s002.docx]

**S1 Table. The distribution of co-infection with multiple pathogens**

| **Pathogens** | **Subjects (n)** |
| --- | --- |
| **Co-infection with virus and bacteria** |  |
| Adenovirus plus: |  |
| *C. pneumoniae* | 3 |
| *C. pneumoniae +* Rhinovirus + Influenza + Coronavirus | 1 |
| *M. pneumoniae* | 1 |
| *K. pneumoniae* | 1 |
| Rhinovirus plus: |  |
| *M. pneumoniae* | 1 |
| Human metapneumovirus |  |
| *M. pneumoniae* | 1 |
| *M. pneumoniae +* Parainfluenza virus | 1 |
| Respiratory syncytial virus |  |
| *M. pneumoniae* | 1 |
| **Co-infection with multiple virus** |  |
| Adenovirus plus: |  |
| Rhinovirus | 16 |
| Rhinovirus + Coronavirus | 3 |
| Rhinovirus + Influenza | 1 |
| Coronavirus | 7 |
| Coronavirus + Human metapneumovirus | 1 |
| Human metapneumovirus | 4 |
| Respiratory syncytial virus | 2 |
| Respiratory syncytial virus + Bocavirus | 1 |
| Influenza | 3 |
| Influenza + Parainfluenza virus | 1 |
| Parainfluenza virus | 2 |
| Rhinovirus plus: |  |
| Human metapneumovirus | 2 |
| Influenza | 1 |
