# Supplementary material for: Clinical Features and Courses of Adenovirus Pneumonia in Healthy Young Adults during an Outbreak among Korean Military Personnel
Source: PLoS One. 2017 Jan 23;12(1):e0170592. doi: 10.1371/journal.pone.0170592 (PMC5256920; doi:10.1371/journal.pone.0170592)
Supplement: S2 Table — (DOCX) [file pone.0170592.s003.docx]

**S2 Table.** Chest computed tomography (CT) scan patterns of adenoviral pneumonia and number of days from fever onset to CT scan (n = 152).

| Chest CT scan patterns | N=152 | Days from fever onset to CT scan |
| --- | --- | --- |
| Focal GGO or minimal consolidation with or without nodules | 37 (24.3) | 2.1 ± 1.1 |
| Segmental GGO with central consolidation | 76 (50.0) | 3.5 ± 1.5 |
| Lobar consolidation with GGO with or without effusion | 15 (9.9) | 4.9 ± 2.0 |
| Bronchopneumonia pattern | 21 (13.8) | 3.7 ± 2.3 |
| Multiple random GGO | 3 (2.0) | 1.7 ± 0.6 |

GGO; Ground-glass opacity
