# Supplementary material for: Clinical Features and Courses of Adenovirus Pneumonia in Healthy Young Adults during an Outbreak among Korean Military Personnel
Source: PLoS One. 2017 Jan 23;12(1):e0170592. doi: 10.1371/journal.pone.0170592 (PMC5256920; doi:10.1371/journal.pone.0170592)
Supplement: S3 Table — (DOCX) [file pone.0170592.s004.docx]

**S3 Table.** Comparison of clinical features associated with severe adenovirus pneumonia and mild to moderate adenovirus pneumonia among military personnel.

| Variables | Severe adenoviral pneumonia  (n=5) | Mild to moderate adenoviral pneumonia  (n=148) | *P* value |
| --- | --- | --- | --- |
| Mean age, year | 20.8 ± 0.4 | 20.1 ± 1.4 | 0.267 |
| Sex, male | 5 (100) | 146 (98.6) | 1.000 |
| Military service period, weeks, median, (IQR) | 8.7 (8.1-15.4) | 7.0 (5.7-8.6) | 0.088 |
| Current smoker | 3 (60.0) | 45 (30.4) | 0.178 |
| Influenza vaccine (< 1 year) | 5 (100) | 140 (94.6) | 1.000 |
| Chronic kidney disease | 0 | 1 (0.7) | 1.000 |
| Asthma | 1 (20.0) | 9 (6.1) | 0.290 |
| Symptoms and signs |  |  |  |
| Cough | 5 (100) | 145 (98.0) | 1.000 |
| Fever | 5 (100) | 146 (98.6) | 1.000 |
| Maximal temperature | 40.0 ± 0.2 | 39.3 ± 0.9 | 0.085 |
| High fever (≥40.0) | 4 (80.0) | 42 (28.4) | 0.029 |
| High fever (≥39.0) | 5 (100) | 111 (75.0) | 0.337 |
| Duration of fever, days | 8.6 ± 1.9 | 6.3 ± 1.6 | 0.002 |
| Sputum production | 5 (100) | 136 (91.9) | 1.000 |
| Purulent sputum (n=141) | 5 (100) | 128 (94.1) | 1.000 |
| Rhinorrhea | 2 (40.0) | 150 (70.9) | 0.160 |
| Nasal congestion | 1 (20.0) | 95 (64.2) | 0.064 |
| Throat clearing | 3 (60.0) | 93 (62.8) | 1.000 |
| Sore throat | 4 (80.0) | 111 (75.0) | 1.000 |
| Pharyngeal inflammation | 5 (100) | 128 (86.5) | 1.000 |
| Blood-tinged sputum | 1 (20.0) | 46 (31.1) | 1.000 |
| Dyspnea or chest discomfort | 4 (80.0) | 28 (18.9) | 0.007 |
| Chest pain | 0 (0) | 27 (18.2) | 0.557 |
| Headache | 5 (100) | 104 (70.3) | 0.332 |
| Diarrhea | 2 (40.0) | 29 (19.6) | 0.267 |
| Myalgia | 5 (100) | 85 (57.4) | 0.078 |
| Wheezing | 2 (40.0) | 0 (0) | 0.001 |
| Crackle | 5 (100) | 76 (51.4) | 0.061 |
| Systemic blood pressure, mm Hg | 116.4 ± 16.9 | 124.9 ± 13.6 | 0.177 |
| Heart rate, beats/min | 91.0 ± 11.4 | 93.9 ± 15.8 | 0.681 |
| Respiratory rate, breaths/min | 33.8 ± 9.5 | 18.1 ± 2.6 | 0.021 |
| Oxygen saturation on room air, % | 89.6 ± 4.7 | 98.0 ± 1.4 | 0.016 |

IQR; interquartile range
