# Supplementary material for: Clinical Features and Courses of Adenovirus Pneumonia in Healthy Young Adults during an Outbreak among Korean Military Personnel
Source: PLoS One. 2017 Jan 23;12(1):e0170592. doi: 10.1371/journal.pone.0170592 (PMC5256920; doi:10.1371/journal.pone.0170592)
Supplement: S4 Table — (DOCX) [file pone.0170592.s005.docx]

**S4 Table.** Comparison of laboratory findings and outcomes associated with severe adenovirus pneumonia and mild to moderate adenovirus pneumonia among military personnel.

| Variables | Severe adenoviral pneumonia  (n=5) | Mild to moderate adenoviral pneumonia  (n=148) | *P* value |
| --- | --- | --- | --- |
| Laboratory findings |  |  |  |
| WBC, 10^9^/L, admission day | 4.48 ± 0.57 | 6.55 ± 3.35 | <0.001 |
| Leukopenia (<4×10^9^/L) | 1 (20.0) | 34 (23.0) | 0.655 |
| Leukocytosis (>10×10^9^/L) | 0 (0) | 19 (12.8) | 1.000 |
| Neutrophil, % | 80.0 ± 7.4 | 67.2 ± 12.4 | 0.023 |
| Lymphocyte, % | 15.7 ± 6.5 | 21.7 ± 11.8 | 0.218 |
| Leukopenia during febrile period | 5 (100) | 91 (61.5) | 0.158 |
| WBC, 10^9^/L, nadir | 3.31 ± 0.45 | 3.91 ± 1.30 | 0.310 |
| Days from fever onset to WBC nadir | 5.6 ± 2.6 | 6.5 ± 1.8 | 0.284 |
| Days from fever onset to the most radiologic aggravation | 9.0 ± 2.7 | 6.3 ± 1.6 | 0.001 |
| Platelet, 10^9^/L | 121 ± 39 | 182 ± 65 | 0.042 |
| Thrombocytopenia (< 150 10^9^/L) | 4 (80.0) | 51 (34.5) | 0.056 |
| Thrombocytopenia during febrile period | 4 (80.0) | 60 (40.5) | 0.162 |
| Hematocrit, % | 42.4 ± 2.5 | 40.7 ± 2.9 | 0.206 |
| CRP, mg/dL | 13.45 ± 3.61 | 6.73 ± 4.74 | 0.002 |
| Procalcitonin, ng/mL (n=117) | 1.08 ± 1.19 | 0.33 ± 0.88 | 0.102 |
| pH | 7.38 ± 0.01 | 7.39 ± 0.04 | 0.893 |
| BUN, mg/dL | 15.3 ± 3.8 | 11.0 ± 3.3 | 0.005 |
| Sodium, mmol/L | 132 ± 4 | 136 ± 3 | 0.003 |
| Glucose, mg/dL | 115 ± 16 | 105 ± 19 | 0.270 |
| Creatine phosphokinase, IU/L (n=127) | 2088 ± 1144 | 746 ± 956 | 0.018 |
| AST, IU/L | 66 ± 32 | 48 ± 48 | 0.430 |
| ALT, IU/L | 23 ± 8 | 33 ± 37 | 0.550 |
| Pneumonia severity on admission |  |  |  |
| PSI score | 67 ± 22 | 28 ± 11 | <0.001 |
| PSI class |  |  | <0.001 |
| Class I | 1 (20.0) | 101 (68.2) |  |
| Class II | 2 (40.0) | 46 (31.1) |  |
| Class III | 2 (40.0) | 1 (0.7) |  |
| Class IV-V | 0 (0) | 0 (0) |  |
| CURB65 |  |  | <0.001 |
| 0 point | 1 (20.0) | 145 (98.0) |  |
| 1 point | 4 (80.0) | 3 (2.0) |  |
| 2-5 points | 0 (0) | 0 (0) |  |

PSI; pneumonia severity index
